# A *post-hoc* analysis of intravitreal aflibercept-treated nAMD patients from ARIES & ALTAIR: predicting treatment intervals and frequency for aflibercept treat-and-extend therapy regimen using machine learning

**DOI:** 10.1007/s00417-025-06812-x

**Published:** 2025-04-10

**Authors:** Matthias Gutfleisch, Britta Heimes-Bussmann, Sökmen Aydin, Ratko Petrovic, Alexander Loktyushin, Masahito Ohji, Kanji Takahashi, Annabelle A. Okada, Paula Scholz, Hossam Youssef, Ulrike Bauer-Steinhusen, Tobias Machewitz, Kai Rothaus, Albrecht Lommatzsch

**Affiliations:** 1 Department of Ophthalmology, St Franziskus-Hospital, Münster, Germany; 2 M3 Macula Monitor Münster GmbH & Co KG, Münster, Germany; 3 deepeye Medical GmbH, Munich, Germany; 4 Shiga University of Medical Science, Seta Tsukinowa-Cho, Otsu, Shiga Japan; 5 School of Medicine, Kansai Medical University, Hirakata City, Osaka, Japan; 6 School of Medicine, Kyorin University, Mitaka-Shi, Tokyo, Japan; 7 Bayer Vital GmbH, Leverkusen, Germany; 8 Bayer Middle East FZE, Dubai, United Arab Emirates; 9 Bayer AG, Berlin, Germany; 10 Department of Ophthalmology, University Duisburg-Essen, Essen, Germany; 11 Achim Wessing Institute of Ophthalmic Diagnostic, University Duisburg-Essen, Essen, Germany

**Keywords:** Neovascular age-related macular degeneration, Anti-VEGF therapy, Treat-and-extend, Artificial intelligence, Transfer learning

## Abstract

**Purpose:**

To predict potential treatment need during treat-and-extend (T&E) anti-vascular endothelial growth factor (VEGF) treatment in neovascular age-related macular degeneration (nAMD) using an artificial intelligence (AI) model trained using transfer learning.

**Methods:**

ARIES and ALTAIR were randomized controlled Phase 3b/4 trials assessing intravitreal aflibercept (IVT-AFL) in patients with nAMD. Following treatment initiation with three monthly injections of IVT-AFL, treatment intervals were re-assessed continuously during the study based on prespecified criteria. In this *post- hoc* analysis, spectral domain optical coherence tomography (SD-OCT) scans from Week (Wk) 8 and Wk 16 visits from patients treated with T&E regimens of 2 mg IVT-AFL over 2 years were utilized to predict individual treatment intervals and frequency. Automated image segmentation of the SD-OCT scans was performed, predictive models of treatment intervals and frequency were developed using machine learning or logistic regression methods, and their performance was evaluated using a fivefold cross-validation. A transfer learning technique was used to adapt existing AI models previously trained on a *pro-re-nata* therapy regimen to the T&E dataset.

**Results:**

In total, 205 ARIES and 112 ALTAIR patient datasets were used for training and evaluation. The following results were achieved with an AI model trained using transfer learning (for ARIES) and logistic regression (for ALTAIR). For prediction of the first treatment interval (short [< 12 weeks] or long [≥ 12 weeks]) following treatment initiation, at Visit 4 (Wk 16), the AI model achieved an area under the receiver operating characteristic curve (AUC) of 0.87 and 0.78 for ARIES and ALTAIR, respectively. For assessment of the individual frequency of IVT-AFL in the first and second study years, the model achieved an AUC of 0.84 and 0.79, respectively, for ARIES, and 0.79 and 0.78, respectively, for ALTAIR. For prediction of the last intended individual treatment interval at the end of Year 2, the AI model achieved an AUC of 0.74 and 0.77 for ARIES and ALTAIR, respectively.

**Conclusion:**

AI trained using transfer learning can be used to predict potential treatment needs for anti-VEGF treatment in nAMD based on SD-OCT scans at Wk 8 and Wk 16, supporting medical decisions on interval adjustments and optimizing individualized IVT-AFL treatment regimens.

**Supplementary Information:**

The online version contains supplementary material available at 10.1007/s00417-025-06812-x.

## Abstract (highlights)

**Key messages:**

*****What is known***:**

- Due to the high treatment burden associated with frequent clinic visits and anti-vascular endothelial growth factor injections required for the treatment of patients with neovascular age-related macular degeneration (nAMD), it would be beneficial to be able to predict potential future treatment need, at initial stages of treatment.

*****What is new***:**

- An artificial intelligence (AI) model, previously trained with a real-world *pro re nata*-treated cohort, can be re-trained and applied to datasets from patients treated with treat-and extend regimens in the clinical study setting to predict potential treatment need.
- AI may be used to predict potential treatment needs for intravitreal aflibercept treatment in nAMD based on optical coherence tomography scans at Weeks 8 and 16, supporting medical decisions on interval adjustments and optimizing individualized treatment regimens.

**Supplementary Information:**

The online version contains supplementary material available at 10.1007/s00417-025-06812-x.

## Introduction

Age-related macular degeneration (AMD) is a leading cause of irreversible vision loss in older adults in the developed world [1]. The prevalence of AMD is expected to rise with the increasing aging population worldwide [1–3], affecting up to 288 million adults in 2040 [1]. The neovascular form of AMD (nAMD) accounts for approximately 10–20% of the total cases of AMD and is responsible for nearly 90% of severe vision loss (20/200 or worse) from AMD [4].

Intravitreal administration of anti-vascular endothelial growth factor (VEGF) agents (including aflibercept, ranibizumab, and faricimab) has substantially improved the prognosis of nAMD and has become the gold standard in the management of the disease [5, 6]. A number of treatment regimens for the intravitreal administration of anti-VEGF agents exist. With the *pro re nata* (PRN) regimen, injections are administered only as needed, based on the detection of disease activity at regular, often monthly, visits [6, 7]. By contrast, in the treat-and-extend (T&E) regimen, an injection is planned to be administered at every scheduled visit, regardless of visual or morphologic status [6]. The interval until the subsequent injection is either maintained or adjusted (increased or decreased) according to functional and morphologic status to maximize injection intervals without disease recurrence [6–8]. Despite superior visual outcomes in multiple prospective clinical trials, the benefit of anti-VEGF therapy is not reflected accordingly in the real world [9–12], with declines in visual acuity and injections often observed over time. The requirement for frequent clinic visits and injections may result in inconsistent dosing regimens or patient nonadherence, with consequent losses of initial treatment benefits, and thus represents a considerable challenge in the routine management of patients with nAMD [13–15]. Therefore, there is a need to optimize the decision process for anti-VEGF treatment of nAMD [12].

The T&E regimen establishes individual treatment intervals following dose modifications over several visits. The ability to predict this adequate treatment interval at the early stages of treatment would be of great benefit to physicians. In clinical studies, adjustment of treatment intervals and monitoring during the T&E regimen are determined by defined functional and optical coherence tomography (OCT) criteria [6]. Through the analysis of digital images (e.g. spectral-domain OCT [SD-OCT]), artificial intelligence (AI) and neural networks can be used to develop algorithms that can support SD-OCT assessments and treatment with anti-VEGF therapy [16]. This includes algorithms that can differentiate presentations of nAMD for subsequent referral [17], assess therapeutic response, and predict anti-VEGF treatment requirements [18–20].

Transfer learning is a powerful technique in machine learning that allows a model to leverage knowledge learned from one task to improve performance on another related task. In particular, transfer learning can mitigate the issue of small sample sizes by utilizing models pretrained on large datasets to extract relevant features that can be fine-tuned on the small dataset [21]. A previous study has described annotation-based deep learning models trained with data from a real-world PRN-treated cohort of patients with nAMD to predict disease activity and the need for injections in the following year of treatment, based on SD-OCT images acquired during treatment initiation [22]. This project aimed to apply transfer learning from this previously PRN-trained AI model to data from prospective, randomized clinical trials that assessed proactive T&E regimens, to predict the potential anti-VEGF treatment requirements of patients with nAMD.

## Methods

### Data

ARIES and ALTAIR were randomized controlled Phase 3b/4 trials assessing proactive T&E regimens in patients with nAMD (ClinicalTrials.gov Identifier: NCT02581891 and NCT02305238) [23, 24] (**Online resource** 1). ARIES was conducted across Europe, North America and Australia, whereas ALTAIR was conducted in Japan. Treatment was initiated with three monthly injections of 2 mg intravitreal aflibercept (IVT-AFL) at Weeks 0, 4, and 8; subsequent treatment intervals were determined at each injection visit over 2 years, based on prespecified SD-OCT criteria (**Online resource** 1 and 2). In routine clinical practice, it is common for clinicians to make treatment decisions for interval adjustment in IVT-AFL treatment regimens based on comparison of an individual’s current SD-OCT scan (also referred to as SD-OCT volume or SD-OCT examination) with the previous one. This was the approach taken for this analysis, where two consecutive SD-OCT scans (at Week [Wk] 8 and Wk 16) were obtained from individual study site databases for both ARIES and ALTAIR and assessed (see additional information on selection of time points in **Online Resource** 3). An additional assessment for the ARIES dataset was also obtained from a reading center (Bern Photographic Reading Center, Bern, Switzerland). Image and treatment data for each patient were linked based on the image acquisition date and the medical record date to generate datasets to train and evaluate AI models to predict expected treatment response after Wk 16.

### Development of AI models

The deepeye® Research tool (https://www.deepeye-medical.com/), an established, OCT device-agnostic modifiable AI platform, was used to evaluate and adapt the AI models. The platform is based on several AI models previously developed and established by the Eye Center at St Franziskus-Hospital, Münster, and deepeye Medical GmbH [17, 22, 25]. Initially, new AI models for each experiment were trained using only T&E datasets from ARIES and ALTAIR i.e. training from scratch (see **Online Resource 3**). Subsequently, a transfer learning technique was used to adapt existing AI models previously trained on the PRN therapy regimen [22] to the T&E datasets from ARIES and ALTAIR.

#### Data preprocessing

**Fig. 1:**
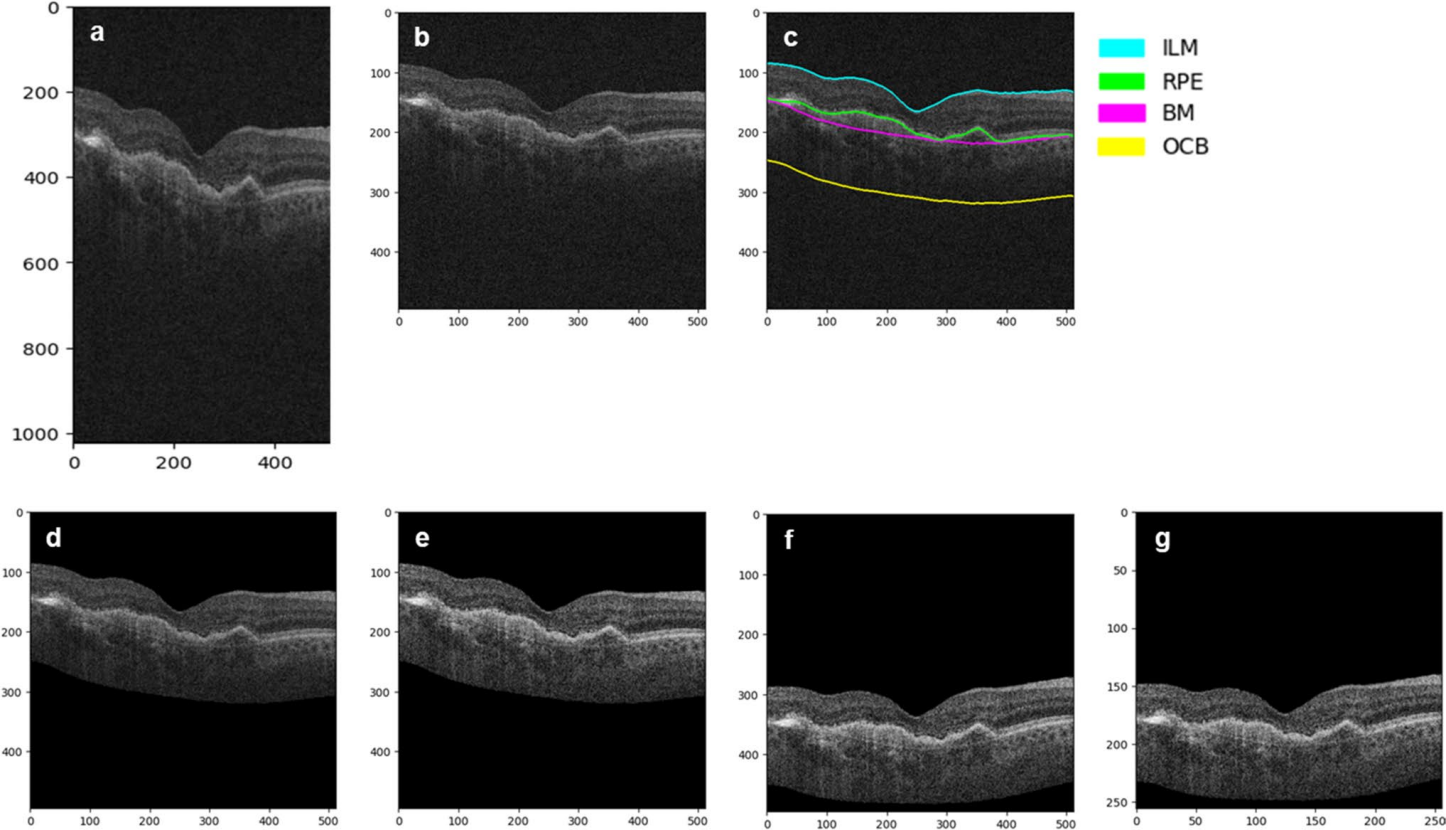
B-scan preprocessing steps. **a**) Original B-scan **b**) Normalization of pixel distances and FoV **c**) AI layer segmentations **d**) Detection of ROI **e**) CLAHE application **f**) Slice alignment **g**) Resized image. AI, artificial intelligence; BM, Bruch’s membrane; CLAHE, contrast-limited adaptive histogram equalization; FoV, field of view; ILM, inner limiting membrane; OCB, outer choroidal boundary; ROI, region of interest; RPE, retinal pigment epithelium

As input for the model, AI-based biomarker segmentation [25] of intraretinal fluid (IRF), subretinal fluid (SRF), inner limiting membrane (ILM), outer plexiform layer (OPL), inner plexiform layer (IPL), and retinal pigment epithelium (RPE) was performed on all SD-OCT scans from ARIES and ALTAIR. Prior to the automatic segmentation, the SD-OCT scans were automatically preprocessed (Fig. 1), as described in **Online Resource** 3. Subsequently, for each processed SD-OCT scan, 28 B-scans around the central B-scan (ensuring an enface Field-of-View of 3.36 mm) were segmented with the AI biomarker model to predict IRF and SRF segmentation masks (**Online Resource** 4A). The segmented B-scans were resized to 128 × 128 pixels and then cropped to a size of 52 × 72 to provide the final 3D-input volume of 28 × 52x72 pixels, which served as input for the deep-learning model.

#### Deep learning model architecture

To build a predictive model of treatment interval and frequency groups, a similar architecture for processing temporally sequential SD-OCT scans [17] was used as a deep learning model (**Online Resource** 4B; details in **Online Resource** 3). A transfer learning technique was also used to adapt the existing AI models previously trained on the PRN therapy regimen [22] to the T&E dataset.

### Model training for deep learning (ARIES)

The performance of the predictive model (assessed as agreement between the AI model and actual study results) was evaluated using fivefold cross-validation at patient level and assessed based on the area under the receiver operating characteristic (ROC) curve. In each of the 5 training iterations, a new rotating subset with 20% of all samples was held out for the test set, ensuring that each sample was classified once as a part of a test set. The remaining samples (80%) were randomly divided into training (64%) and validation (16%) sets. Additional details are provided in **Online Resource 3**.

### Model training using logistic regression (ALTAIR)

Results using the ARIES dataset were sufficiently performant (i.e. high scores for accuracy, specificity, and sensitivity that are significantly above random chance), but the model was found to overfit in the case of the ALTAIR dataset. Instead, a logistic regression model (based on a binary classification machine learning method using a non-linear activation function) was used for the ALTAIR dataset, as described in **Online Resource** 3.

### Statistical analysis

Model assessments were based on the following parameters: accuracy, the number of correct predictions divided by the total prediction number; sensitivity, the model’s ability to predict true positives of each available category; specificity, the model's ability to predict true negatives of each available category; and balanced accuracy, calculated by (sensitivity + specificity) / 2. The closer the balanced accuracy is to 1, the better the model is able to classify observations correctly.

The overall performance of each model was assessed based on the area under the ROC curve (AUC), which plots the relationship between the true positive- and false positive rate (plotted as a mean of 5 × fivefold cross-validation). The AUC score ranges from 0 to 1, with 1 indicating a perfect classifier and 0.5 representing a classifier without discriminative power. The model performance is summarized by the sensitivity and specificity at an optimal operating point based on the equal error rate, where the false-negative rate and false-positive rate are equivalent.

### Validation of IRF and SRF biomarkers

As the prediction of the AI algorithm is based on the correct detection of SRF and IRF in SD-OCT scans, an initial validation of the detection of both fluid biomarkers was performed to assess how well automated image segmentation matched actual clinician determination from ARIES (study sites and reading center).

### Experiments

Experiments were carried out to assess agreement between the AI model and actual study results, utilizing segmented biomarkers of IRF and SRF from Wk 8 and Wk 16 as input.

#### Experiment 1: Prediction of the first treatment interval decision after treatment initiation

This experiment aimed to predict the first potential adequate individual treatment interval decision after treatment initiation, i.e. at Visit 4 (Wk 16). As data were only available for observed (but not assigned) treatment intervals following Visit 4 in ARIES and ALTAIR, extend/non-extend decisions in the first four visits following treatment initiation (i.e. Visits 4, 5, 6, and 7) were used instead for establishing the ground truth at Visit 7, which was then extrapolated to Visit 4 (see details in **Online Resource** 3). Statistical distribution of observed injection intervals at Visit 7 established the ground truth that a short interval would correspond to < 12 weeks and a long interval would correspond to ≥ 12 weeks.

#### Experiment 2: Prediction of treatment need for IVT-AFL in Year 1 and Year 2

In this experiment, the ground truth was defined as follows: requirements for IVT-AFL were defined as low (< 8 injections) or high (≥ 8 injections) in the first year, and as low (< 5 injections) or high (≥ 5 injections) in the second year. The 8-injection cut-off was chosen based on the analogous regimen of fixed dosing of 2 mg every 8 weeks in pivotal phase 3 trials for aflibercept (VIEW 1 and VIEW 2) [26], in which the participants received slightly more than 7 injections in the first year (3 monthly and 4–5 bi-monthly follow-up treatments) [26]. Thus, a requirement for 8 or more injections in Year 1 would be considered to be a high treatment need.

#### Experiment 3: Prediction of IVT-AFL treatment interval at the end of Year 2 of study

In this experiment, the AI algorithm was trained to predict the last individual treatment interval as defined in the study database: short (< 12 weeks) versus long (≥ 12 weeks).

## Results

### Data

SD-OCT data were obtained from 224 of 236 patients who completed the ARIES study, and 112 of 246 patients who completed the ALTAIR study. The low number of records from ALTAIR was due to the limited number of SD-OCT scans that were only obtained retrospectively from some study sites as no reading centers were involved in this study. As ARIES and ALTAIR were multisite studies, a variety of devices were used to obtain the SD-OCT scans (**Online Resource** 5). After preprocessing and removing patient records with missing or incomplete data, 205 ARIES and 112 ALTAIR patient datasets (comprising 2003 and 1339 SD-OCT scans, respectively) were used for training.

### Validation of IRF and SRF

#### ARIES

Two sets of data based on the ARIES OCT scans were obtained, one from the study sites and another from the reading center. In ARIES, the anatomic criteria for extending the treatment intervals, based on SD-OCT, included the absence of IRF, and SRF not exceeding 50 µm in height. IRF was graded according to its presence or absence, and assessment of this biomarker demonstrated a better agreement between AI vs reading center evaluation in comparison to study site vs reading center evaluation (Table 1). The threshold for SRF height was defined as 50 µm by study protocol. Due to the fixed threshold, the AI performed less well than the study site for assessment of SRF height (Table 1). Threshold values for the presence of IRF and SRF were optimized to provide the best agreement between AI and reading center biomarker assessments. These values were based on the equal error rate (EER), so that the false positive rate (FPR) and false negative rate (FNR) are equal. The threshold values were computed as 6.0 nL for the presence of IRF and 16 nL for the presence of SRF. The achieved balanced accuracy against the reading center was 0.75 (study site) vs 0.86 (AI) for IRF presence, 0.81 (study site) vs 0.79 (AI) for SRF height ≥ 50 µm, and 0.85 (AI) for SRF presence (**Online Resource** 6). AI outperformed the study site for IRF presence but not for SRF height, due to the fixed SRF height threshold based on the study protocol. These results indicated that the AI biomarker model results overall conformed more (i.e. IRF presence: much better; SRF height: almost similar) with results from the reading center than the study site datasets, likely because the SD-OCT scans at the reading center were consistently assessed by expert graders.

**Table 1:** Confusion matrices between study site vs reading center and AI vs reading center for IRF presence, SRF height, and SRF presence

		**IRF presence**		**SRF height ≥ 50 µm**		**SRF presence**	
		Study reading center		Study reading center		Study reading center	
		**No**	**Yes**	**No**	**Yes**	**No**	**Yes**
Study site	**No**	984	30	1063	143	-*	-
	**Yes**	582	208	153	414	-	-
AI	**No**	1340	35	731	9	812	138
	**Yes**	226	221	485	548	144	728

*No labelling for ‘SRF presence’ was carried out at study sites. AI, artificial intelligence; IRF, intraretinal fluid; SRF, subretinal fluid

#### ALTAIR

No reading center grading of IRF and SRF was available for ALTAIR.

### Experiment 1: Prediction of the first treatment interval decision after treatment initiation

This model aimed to predict the treatment interval (short or long) for a patient at Visit 4 (Wk 16) based on SD-OCT scans from Wk 8 and Wk 16.

#### ARIES

**Fig. 2:**
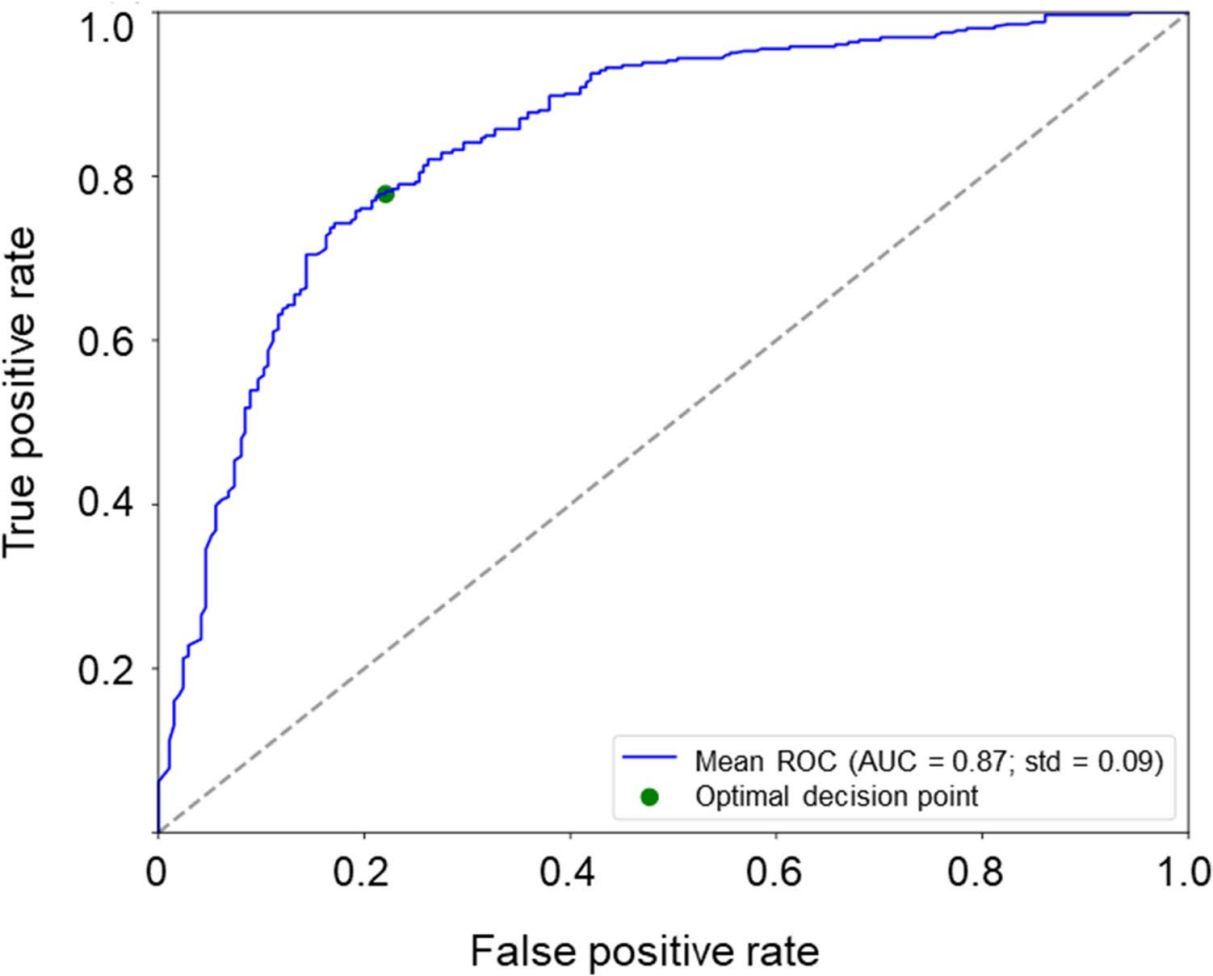
Experiment 1: Receiver operating characteristic curve showing runs from 5 × fivefold cross-validation following transfer learning (ARIES). AUC, area under the ROC curve; ROC, receiver operating characteristic; std, standard deviation

Only the inputs of the early-start T&E study arm were used in this experiment (108 patients). Training from scratch and transfer learning were conducted separately, and corresponding results were compared. The AI model trained from scratch achieved 91% sensitivity, 38% specificity and 64% accuracy, with an AUC of 0.77 (Table 2). A significant improvement in specificity (from 38 to 71%) and overall accuracy (from 64 to 77%) was seen with the AI model after applying transfer learning compared with the AI model trained from scratch. The AI algorithm predicted treatment intervals at Visit 4, with 83% sensitivity and 71% specificity resulting in an overall accuracy of 77%, and achieved an improved AUC of 0.87 following transfer learning (Table 2 and Fig. 2).

**Table 2:** ARIES Algorithm results for Experiments 1, 2, and 3

**EXPERIMENT 1**^**a**^						
	**AUC**	**Accuracy**	**Sensitivity**	**Specificity**	**Patients in Group A**^**b**^ (< 3 extension decisions taken at Visits 4, 5, 6, and 7)	**Patients in Group B**^**b**^ (3 or 4 extension decisions taken at Visits 4, 5, 6, and 7)
**Training from scratch**	0.77	64%	91% (66/72)	38% (14/36)	72	36
**Following transfer learning**	0.87	77%	83% (60/72)	71% (26/36)	72	36
**EXPERIMENT 2**^**a**^						
	**AUC**	**Accuracy**	**Sensitivity**	**Specificity**	**Patients with high IVT-AFL need**^**c**^	**Patients with low IVT-AFL need**^**c**^
**YEAR 1**						
**Training from scratch**	0.77	63%	84% (54/64)	41% (18/44)	64	44
**Following transfer learning**	0.84	75%	81% (52/64)	70% (31/44)	64	44
**YEAR 2**						
**Training from scratch**	0.78	71%	82% (86/105)	59% (68/116)	105	116
**Following transfer learning**	0.79	73%	75% (79/105)	71% (82/116)	105	116
**EXPERIMENT 3**^**a**^						
	**AUC**	**Accuracy**	**Sensitivity**	**Specificity**	**Patients with short intervals (< 12 weeks) at end of Year 2**	**Patients with long intervals (≥ 12 weeks) at end of Year 2**
**Training from scratch**	0.74	67%	83% (96/116)	52% (55/105)	116	105
**Following transfer learning**	0.74	71%	80% (93/116)	63% (66/105)	116	105

^**a**^The numerator and the denominator of the fractions for sensitivity and specificity are mean values of the fivefold cross-validation; as such, the result of division may not correspond exactly to the percentage value specified

^**b**^Group A: predicted ‘short’ interval at Visit 7; Group B: predicted ‘long’ interval at Visit 7

^**c**^High IVT-AFL need was defined as ≥ 8 and ≥ 5 IVT-AFL injections per year for Year 1 and 2, respectively; low IVT-AFL need was defined as < 8 and < 5 IVT-AFL injections per year for Year 1 and 2, respectively

AUC, area under the curve; IVT-AFL, intravitreal aflibercept

#### ALTAIR

Application of the AI model to the ALTAIR dataset (*n* = 83) predicted the initial injection interval at Visit 4 with 85% sensitivity and 71% specificity, with an overall accuracy of 78%; the model achieved an AUC of 0.78 (Table 3).

**Table 3:** ALTAIR: Algorithm results for Experiments 1, 2, and 3

**EXPERIMENT 1**^**a**^						
	**AUC**	**Accuracy**	**Sensitivity**	**Specificity**	**Patients in Group A**^**b**^ (< 3 extension decisions taken at Visits 4, 5, 6, and 7)	**Patients in Group B**^**b**^ (3 or 4 extension decisions taken at Visits 4, 5, 6, and 7)
	0.78	78%	85% (31/37)	71% (36/46)	37	46
**EXPERIMENT 2**^**a**^						
	**AUC**	**Accuracy**	**Sensitivity**	**Specificity**	**Patients with high IVT-AFL need**^**c**^	**Patients with low IVT-AFL need**^**c**^
**Year 1**	0.79	79%	79% (27/35)	78% (42/54)	35	54
**Year 2**	0.78	78%	87% (34/39)	69% (26/38)	39	38
**EXPERIMENT 3**^**a**^						
	**AUC**	**Accuracy**	**Sensitivity**	**Specificity**	**Patients with short intervals (< 12 weeks) at end of Year 2**	**Patients with long intervals (≥ 12 weeks) at end of Year 2**
	0.77	76%	81% (34/43)	71% (24/34)	43	34

^**a**^The numerator and the denominator of the fractions for sensitivity and specificity are mean values of the fivefold cross-validation; as such, the result of division may not correspond exactly to the percentage value specified

^**b**^Group A: predicted ‘short’ interval at Visit 7; Group B: predicted ‘long’ interval at Visit 7

^**c**^High IVT-AFL need was defined as ≥ 8 and ≥ 5 IVT-AFL injections per year for Year 1 and 2, respectively; low IVT-AFL need was defined as < 8 and < 5 IVT-AFL injections per year for Year 1 and 2, respectively

AUC, area under the curve; IVT-AFL, intravitreal aflibercept

### Experiment 2: Prediction of treatment need in study Years 1 and 2

This model aimed to predict the frequency of IVT-AFL injections in the first and second year of treatment.

#### ARIES

**Fig. 3:**
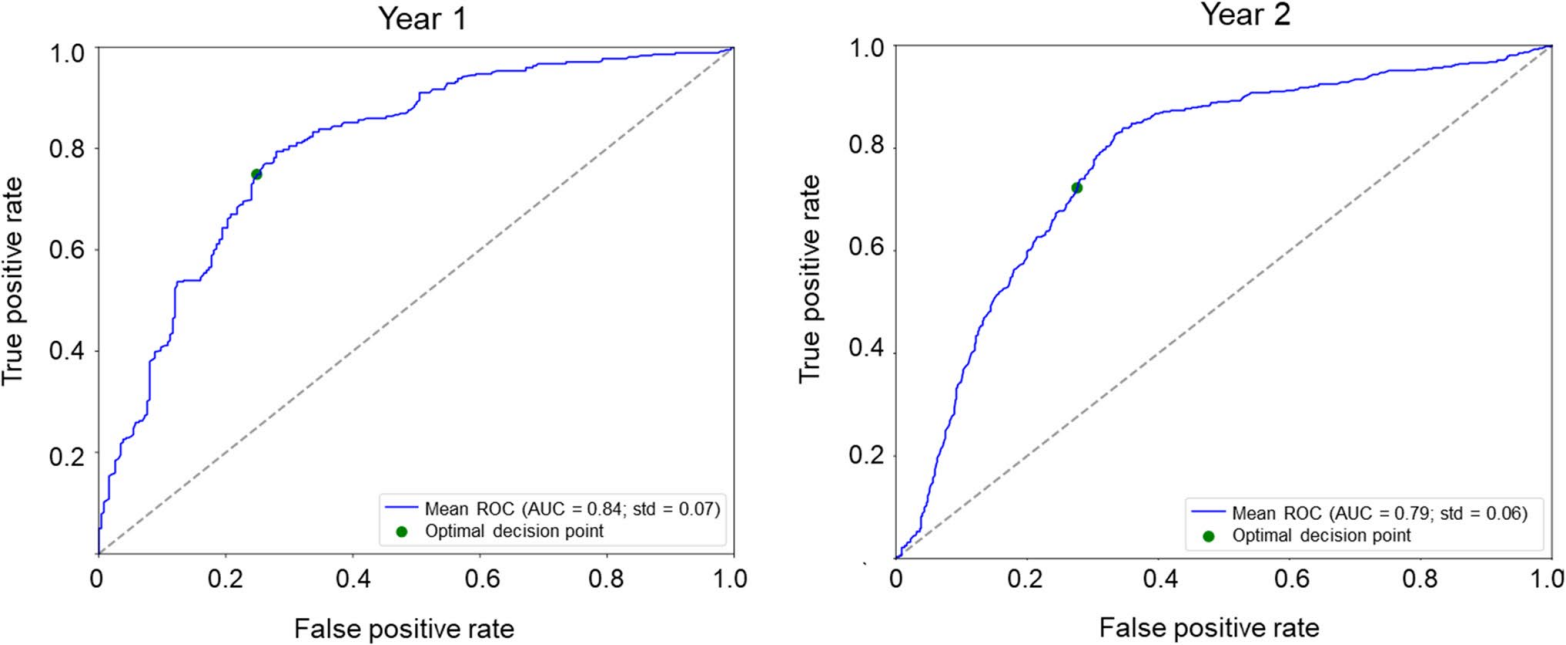
Experiment 2: Receiver operating characteristic curves showing runs from 5 × fivefold cross-validation for Year 1 and Year 2 following transfer learning (ARIES). AUC, area under the ROC curve; ROC, receiver operating characteristic; std, standard deviation

For the Year 1 analysis, data from 108 patients from the ‘early-start’ T&E study arm were used. The results showed that there was a significant improvement in the specificity (from 41 to 70%) and overall accuracy (from 63 to 70%) following transfer learning (Table 2), with 81% sensitivity. The overall accuracy of the algorithm in this case was 75%. For Year 2, results from 221 patients from both the ‘early-start’ and ‘late-start’ T&E study arms were used and the overall accuracy of the algorithm following transfer learning was 73% (Table 2). The model achieved AUCs of 0.78 and 0.79 for Year 1 and 2, respectively (Table 2 and Fig. 3).

#### ALTAIR

Data from 89 patients were used for the Year 1 analysis. The algorithm demonstrated a sensitivity of 79% and specificity of 78%, with an overall accuracy of 79% (Table 3). Data from 77 patients were used for the Year 2 analysis; the algorithm demonstrated a sensitivity of 87% and a specificity of 69%, with an overall accuracy of 78%. The model was able to achieve an AUC of 0.79 and 0.78 for prediction of frequency of IVT-AFL injections in the first and second year of treatment of ALTAIR, respectively (Table 3).

### Experiment 3: Prediction of IVT-AFL treatment interval at the end of Year 2 of study

#### ARIES

**Fig. 4:**
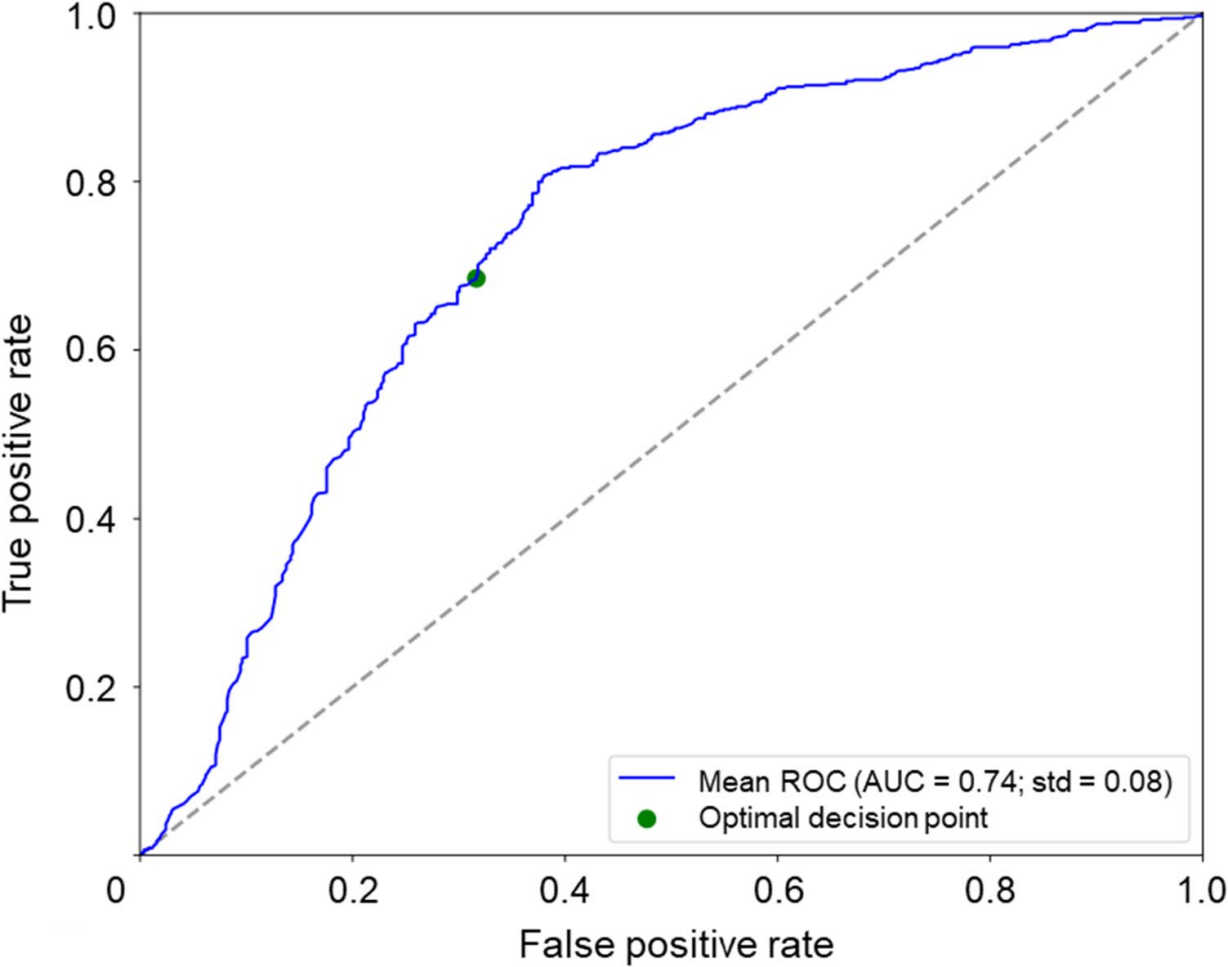
Experiment 3: Receiver operating characteristic curve showing runs from 5 × fivefold cross-validation following transfer learning, threshold 12 weeks (ARIES). AUC, area under the ROC curve; ROC, receiver operating characteristic; std, standard deviation

Data from 221 patients from both the ‘early-start’ and ‘late-start’ T&E study arms were used. Following transfer learning, the overall accuracy of the algorithm improved from 67 to 71% and predicted treatment intervals at the end of Year 2 with 80% sensitivity and 63% specificity (Table 2). In this case, the AUC (0.74) remained the same before and after transfer learning (Table 2 and Fig. 4).

#### ALTAIR

Data from 77 patients were used for this experiment and the overall accuracy of the algorithm in this case was 76%. The model predicting the last treatment interval at the end of Year 2 in ALTAIR achieved an AUC of 0.77 (Table 3).

## Discussion

Patients undergoing anti-VEGF treatment as part of a T&E regimen may have their individual dosing intervals extended during treatment. In daily practice, ophthalmologists face challenges in predicting the expected treatment intervals for individual patients at the end of the initial monthly doses. In recent years, a number of studies have focused on predicting anti-VEGF treatment requirements from longitudinal retinal OCT imaging based on scans acquired during the treatment initiation phase. This includes the use of machine learning models to predict anti-VEGF treatment demand in patients with retinal diseases treated according to a T&E regimen [27], and visual outcomes and treatment needs in T&E [20] and PRN regimens [22, 28] in nAMD. Explainable AI systems that can derive clinically meaningful features from OCT B-scan images to differentiate between normal eyes, those with different grades of AMD, and non-AMD diseases, have also been developed [29].

In this study, automated AI-based OCT quantification was performed for imaging biomarkers associated with nAMD disease progression. We showed that AI models can predict individual treatment needs for a high proportion of patients with nAMD from the ARIES and ALTAIR studies based on imaging biomarkers from OCT examinations at Wk 8 and Wk 16, demonstrating that these are able to provide a good indication of treatment needs at later time points. This was also previously shown in a *post-hoc* analysis of the ALTAIR study [30], where fluid outcomes at Wk 16 were predictive of best-corrected visual acuity outcomes at the end of the study at 96 weeks. The time points were chosen as there were indications from previous analyses that they not only appear to be clinically meaningful, but also have a good prognostic value.

### ARIES

The analysis of the ARIES dataset showed that transfer learning led to better overall model performance compared to training from scratch. The algorithm used for transfer learning was originally trained on PRN data and appeared to be able to extract useful features that could be generalized to the T&E dataset. Models trained using transfer learning achieved an AUC of 0.87 for predicting the first potentially adequate individual treatment interval after treatment initiation (Experiment 1) and 0.74 for predicting the last treatment interval at the end of a 2-year regimen (Experiment 3). These models also achieved AUCs of 0.84 and 0.79, respectively, for predicting the individual frequency of IVT-AFL in the first and second years of study (Experiment 2). Across all experiments, the algorithm's sensitivity and specificity were generally better balanced with transfer learning. In comparison to results from the ‘training from scratch’ approach, the AUC following transfer learning was superior in Experiments 1 and 2 and of the same quality in Experiment 3. These results demonstrate that well-trained PRN models can be successfully adapted to other treatment strategies through transfer learning. When comparing Experiment 1 and 3, it was noticeable that the shorter the time to the endpoint, the more accurate the prediction.

### ALTAIR

In the ALTAIR analysis, the initial AI models (trained from scratch and transfer learning) were found to overfit the dataset, likely due to a combination of factors. It is important to note that it was only possible to obtain 50% of the data points from ALTAIR. When training with a small number of data points, there is a risk of overfitting, especially when using high-capacity methods such as deep learning, as done with the ARIES dataset. Additionally, the lack of additional verification of labels from the reading center resulted in inconsistencies and inaccuracies, as observed during data quality re-evaluation runs (data not shown). Also, unlike ARIES, ALTAIR data were obtained using a wider range of OCT devices, contributing to inconsistencies, resulting in a more heterogeneous data pool. However, the results show that logistic regression can also predict the treatment intervals and frequency with good accuracy. As the logistic regression is based on the annotation of the AI segmentation algorithm (see **Online Resource** 4), it can be concluded that the original algorithm provides sufficiently good annotation performance, even when applied to this extended dataset used in this study.

### Limitations

This study has some limitations that are inherent to *post-hoc* analyses. The analysis was based on data from controlled studies, with treatment decisions based on a predefined protocol and defined parameters as assessed by study clinicians. Furthermore, the OCT images were not specifically acquired for this analysis. The data were obtained from a preselected cohort of patients, which may not fully reflect real-world situations, and may affect the generalizability of our findings. Cross-validation was required due to the small sample size; as such, external validation on an independent test set is required to confirm the generalizability of the models. It is acknowledged that a significantly larger sample size would be required to generate clinically applicable models. Nonetheless, it should be noted that these experiments are comparable in terms of the small sample size (N < 250) to a previous publication (Bogunovich et al. 2022) that examined similar datasets and endpoints [20].

## Conclusions

This proof-of-concept study demonstrates that AI has the potential to support decision-making for interval adjustment in individualized IVT-AFL treatment regimens, despite variability in OCT assessments from different sources. However, studies with larger datasets will be required in order to confirm the findings of this study. Future research could investigate whether statistical methods applied according to segmentation models can be used to gain insights into the influence of individual biomarkers, such as SRF and IRF on treatment intensity needs. Further quantification of biomarkers, including volume, area, and localization within the Early Treatment Diabetic Retinopathy Screening Study grid, could enhance the precision of treatment strategies and improve patient outcomes.

## Supplementary Information

Below is the link to the electronic supplementary material.

Supplementary file1 (DOCX 610 KB)

## Abbreviations

AI: Artificial intelligence
AMD: Age-related macular degeneration
AUC: Area under the curve
BM: Bruch’s membrane
CLAHE: Contrast-limited adaptive histogram equalization
CNN: Convolutional neural network
EER: Equal error rate
FPR: False positive rate
FNR: False negative rate
FoV: Field of view
GAP: Global average pooling
ILM: Internal limiting membrane
IPL: Inner plexiform layer
IRF: Intraretinal fluid
IVT-AFL: Intravitreal aflibercept
LSTM: Long short-term memory
nAMD: Neovascular age-related macular degeneration
OCB: Outer choroidal boundary
OCT: Optical coherence tomography
OPL: Outer plexiform layer
PRN: *Pro re* *nata*
ROC: Receiver operating characteristic
ROI: Region of interest
RPE: Retinal pigment epithelium
SD-OCT: Spectral-domain optical coherence tomography
SRF: Subretinal fluid
T&E: Treat-and-extend
VEGF: Vascular endothelial growth factor
Wk: Week

## References

[1] Wong WL, Su X, Li X, Cheung CM, Klein R, Cheng CY, Wong TY (2014) Global prevalence of age-related macular degeneration and disease burden projection for 2020 and 2040: a systematic review and meta-analysis. Lancet Glob Health 2:e106-116. 10.1016/S2214-109X(13)70145-1 25104651 10.1016/S2214-109X(13)70145-1

[2] Korb CA, Elbaz H, Schuster AK, Nickels S, Ponto KA, Schulz A, Wild PS, Munzel T, Beutel ME, Schmidtmann I, Lackner KJ, Peto T, Pfeiffer N (2022) Five-year cumulative incidence and progression of age-related macular degeneration: results from the German population-based Gutenberg Health Study (GHS). Graefes Arch Clin Exp Ophthalmol 260:55–64. 10.1007/s00417-021-05312-y 34424371 10.1007/s00417-021-05312-y PMC8763742

[3] Smith W, Assink J, Klein R, Mitchell P, Klaver CC, Klein BE, Hofman A, Jensen S, Wang JJ, de Jong PT (2001) Risk factors for age-related macular degeneration: Pooled findings from three continents. Ophthalmology 108:697–704. 10.1016/s0161-6420(00)00580-7 11297486 10.1016/s0161-6420(00)00580-7

[4] Ferris FL 3rd, Fine SL, Hyman L (1984) Age-related macular degeneration and blindness due to neovascular maculopathy. Arch Ophthalmol 102:1640–1642. 10.1001/archopht.1984.01040031330019 6208888 10.1001/archopht.1984.01040031330019

[5] Schmidt-Erfurth U, Chong V, Loewenstein A, Larsen M, Souied E, Schlingemann R, Eldem B, Mones J, Richard G, Bandello F, European Society of Retina Specialists (2014) Guidelines for the management of neovascular age-related macular degeneration by the European Society of Retina Specialists (EURETINA). Br J Ophthalmol 98:1144–1167. 10.1136/bjophthalmol-2014-305702 25136079 10.1136/bjophthalmol-2014-305702 PMC4145443

[6] Lanzetta P, Loewenstein A, Vision Academy Steering Committee (2017) Fundamental principles of an anti-VEGF treatment regimen: optimal application of intravitreal anti-vascular endothelial growth factor therapy of macular diseases. Graefes Arch Clin Exp Ophthalmol 255:1259–1273. 10.1007/s00417-017-3647-4 28527040 10.1007/s00417-017-3647-4 PMC5486551

[7] Freund KB, Korobelnik JF, Devenyi R, Framme C, Galic J, Herbert E, Hoerauf H, Lanzetta P, Michels S, Mitchell P, Mones J, Regillo C, Tadayoni R, Talks J, Wolf S (2015) Treat-and-extend regimens with anti-VEGF agents in retinal diseases: A Literature Review and Consensus Recommendations. Retina 35:1489–1506. 10.1097/IAE.0000000000000627 26076215 10.1097/IAE.0000000000000627

[8] Okada M, Kandasamy R, Chong EW, McGuiness M, Guymer RH (2018) The treat-and-extend injection regimen versus alternate dosing strategies in age-related macular degeneration: A systematic review and meta-analysis. Am J Ophthalmol 192:184–197. 10.1016/j.ajo.2018.05.026 29885297 10.1016/j.ajo.2018.05.026

[9] Mehta H, Kim LN, Mathis T, Zalmay P, Ghanchi F, Amoaku WM, Kodjikian L (2020) Trends in real-world neovascular AMD treatment outcomes in the UK. Clin Ophthalmol 14:3331–3342. 10.2147/OPTH.S275977 33116384 10.2147/OPTH.S275977 PMC7569079

[10] Khanani AM, Skelly A, Bezlyak V, Griner R, Torres LR, Sagkriotis A (2020) SIERRA-AMD: A retrospective, real-world evidence study of patients with neovascular age-related macular degeneration in the United States. Ophthalmol Retina 4:122–133. 10.1016/j.oret.2019.09.009 31812631 10.1016/j.oret.2019.09.009

[11] Kiss S, Campbell J, Almony A, Shih V, Serbin M, LaPrise A, Wykoff CC (2020) Management and outcomes for neovascular age-related macular degeneration: Analysis of United States electronic health records. Ophthalmology 127:1179–1188. 10.1016/j.ophtha.2020.02.027 32345477 10.1016/j.ophtha.2020.02.027

[12] Vogt D, Deiters V, Herold TR, Guenther SR, Kortuem KU, Priglinger SG, Wolf A, Schumann RG (2022) Optimal patient adherence and long-term treatment outcomes of neovascular age-related macular degeneration in real-life. Curr Eye Res 47:889–896. 10.1080/02713683.2022.2044056 35179427 10.1080/02713683.2022.2044056

[13] Ehlken C, Helms M, Bohringer D, Agostini HT, Stahl A (2018) Association of treatment adherence with real-life VA outcomes in AMD, DME, and BRVO patients. Clin Ophthalmol 12:13–20. 10.2147/OPTH.S151611 29339917 10.2147/OPTH.S151611 PMC5745150

[14] Boulanger-Scemama E, Sayag D, Ha Chau Tran T, Quaranta-El Maftouhi M, Rumen F, Creuzot-Garcher C, Blanco Garavito R, Jung C, Souied E (2016) Ranibizumab and exudative age-related macular degeneration: 5-year multicentric functional and anatomical results in real-life practice. J Fr Ophtalmol 39:668–674. 10.1016/j.jfo.2016.06.001 27609025 10.1016/j.jfo.2016.06.001

[15] Shahzad H, Mahmood S, McGee S, Hubbard J, Haque S, Paudyal V, Denniston AK, Hill LJ, Jalal Z (2023) Non-adherence and non-persistence to intravitreal anti-vascular endothelial growth factor (anti-VEGF) therapy: a systematic review and meta-analysis. Syst Rev 12:92. 10.1186/s13643-023-02261-x 37269003 10.1186/s13643-023-02261-x PMC10237080

[16] Muntean GA, Marginean A, Groza A, Damian I, Roman SA, Hapca MC, Muntean MV, Nicoara SD (2023) The predictive capabilities of artificial intelligence-based OCT analysis for age-related macular degeneration progression-a systematic review. Diagnostics (Basel) 13 10.3390/diagnostics13142464 10.3390/diagnostics13142464 PMC10378064 37510207

[17] Gutfleisch M, Ester O, Aydin S, Quassowski M, Spital G, Lommatzsch A, Rothaus K, Dubis AM, Pauleikhoff D (2022) Clinically applicable deep learning-based decision aids for treatment of neovascular AMD. Graefes Arch Clin Exp Ophthalmol 260:2217–2230. 10.1007/s00417-022-05565-1 35064365 10.1007/s00417-022-05565-1

[18] Schmidt-Erfurth U, Mulyukov Z, Gerendas BS, Reiter GS, Lorand D, Weissgerber G, Bogunovic H (2023) Therapeutic response in the HAWK and HARRIER trials using deep learning in retinal fluid volume and compartment analysis. Eye (Lond) 37:1160–1169. 10.1038/s41433-022-02077-4 35523860 10.1038/s41433-022-02077-4 PMC10101971

[19] Bogunovic H, Waldstein SM, Schlegl T, Langs G, Sadeghipour A, Liu X, Gerendas BS, Osborne A, Schmidt-Erfurth U (2017) Prediction of anti-VEGF treatment requirements in neovascular AMD using a machine learning approach. Invest Ophthalmol Vis Sci 58:3240–3248. 10.1167/iovs.16-21053 28660277 10.1167/iovs.16-21053

[20] Bogunovic H, Mares V, Reiter GS, Schmidt-Erfurth U (2022) Predicting treat-and-extend outcomes and treatment intervals in neovascular age-related macular degeneration from retinal optical coherence tomography using artificial intelligence. Front Med (Lausanne) 9:958469. 10.3389/fmed.2022.958469 36017006 10.3389/fmed.2022.958469 PMC9396241

[21] Zhuang F (2021) A comprehensive survey on transfer learning. Proc IEEE 1:43–76

[22] Rothaus K, Heimes-Bussmann B, Aydin S, Quassowski M, Ziegler M, Lange C, Spital G, Lommatzsch A, Gutfleisch M (2022) Prädiktion des Therapiebedarfs bei neovaskulärer AMD (nAMD) mittels annotationsbasierter Deep-Learning-Modelle. DOG (German Society of Ophtalmology) Congress, Berlin.

[23] Mitchell P, Holz FG, Hykin P, Midena E, Souied E, Allmeier H, Lambrou G, Schmelter T, Wolf S (2021) Efficacy and safety of intravitreal efficacy using a treat- and-extend regimen for neovascular age-related macular degeneration: The ARIES study: A randomized clinical trial. Retina 41:1911–1920. 10.1097/IAE.0000000000003128 33782365 10.1097/IAE.0000000000003128 PMC8384251

[24] Ohji M, Takahashi K, Okada AA, Kobayashi M, Matsuda Y, Terano Y (2020) Efficacy and safety of intravitreal aflibercept treat-and-extend regimens in exudative age-related macular degeneration: 52- and 96-week findings from ALTAIR : A randomized controlled trial. Adv Ther 37:1173–1187. 10.1007/s12325-020-01236-x 32016788 10.1007/s12325-020-01236-x PMC7089719

[25] Gutfleisch M, Heimes-Bussmann B, Aydin S, Faatz P, Kintzinger K, Spickermann L, Tieck J, Koch H, Oehlschläger J, Ziegler M, Pauleikhoff D, Lange C, Spital G, Lommatzsch A, Rothaus K (2022) Annotation von SD-OCT-Biomarkern bei nAMD zur Entwicklung erklärbarer KI-Modelle (XAI)DOG (German Society of Ophtalmology) Congress, Berlin.

[26] Heier JS, Brown DM, Chong V, Korobelnik JF, Kaiser PK, Nguyen QD, Kirchhof B, Ho A, Ogura Y, Yancopoulos GD, Stahl N, Vitti R, Berliner AJ, Soo Y, Anderesi M, Groetzbach G, Sommerauer B, Sandbrink R, Simader C, Schmidt-Erfurth U, VIEW 1 and VIEW 2 Study Groups (2012) Intravitreal aflibercept (VEGF trap-eye) in wet age-related macular degeneration. Ophthalmology 119:2537–2548. 10.1016/j.ophtha.2012.09.006 23084240 10.1016/j.ophtha.2012.09.006

[27] Gallardo M, Munk MR, Kurmann T, De Zanet S, Mosinska A, Karagoz IK, Zinkernagel MS, Wolf S, Sznitman R (2021) Machine learning can predict anti-VEGF treatment demand in a treat-and-extend regimen for patients with neovascular AMD, DME, and RVO associated macular edema. Ophthalmol Retina 5:604–624. 10.1016/j.oret.2021.05.002 33971352 10.1016/j.oret.2021.05.002

[28] Romo-Bucheli D, Erfurth US, Bogunovic H (2020) End-to-end deep learning model for predicting treatment requirements in neovascular AMD from longitudinal retinal OCT imaging. IEEE J Biomed Health Inform 24:3456–3465. 10.1109/JBHI.2020.3000136 32750929 10.1109/JBHI.2020.3000136

[29] Elsharkawy M, Sharafeldeen A, Khalifa F, Soliman A, Elnakib A, Ghazal M, Sewelam A, Thanos A, Sandhu HS, El-Baz A (2024) A clinically explainable AI-based grading system for age-related macular degeneration using optical coherence tomography. IEEE J Biomed Health Inform PP 10.1109/JBHI.2024.3355329 10.1109/JBHI.2024.3355329 38231804

[30] Ohji M, Okada AA, Sasaki K, Moon SC, Machewitz T, Takahashi K, ALTAIR Investigators (2021) Relationship between retinal fluid and visual acuity in patients with exudative age-related macular degeneration treated with intravitreal aflibercept using a treat-and-extend regimen: subgroup and post-hoc analyses from the ALTAIR study. Graefes Arch Clin Exp Ophthalmol 259:3637–3647. 10.1007/s00417-021-05293-y 34283294 10.1007/s00417-021-05293-y PMC8589769

