# Supplementary material for: A post-hoc analysis of intravitreal aflibercept-treated nAMD patients from ARIES & ALTAIR: predicting treatment intervals and frequency for aflibercept treat-and-extend therapy regimen using machine learning
Source: Graefes Arch Clin Exp Ophthalmol. 2025 Apr 10;263(7):1885–97. doi: 10.1007/s00417-025-06812-x (PMC12373702; doi:10.1007/s00417-025-06812-x)
Supplement: Supplementary file 1 — Supplementary file1 (DOCX 610 KB) [file 417_2025_6812_MOESM1_ESM.docx]

**SUPPLEMENTARY INFORMATION (ONLINE RESOURCE)**

**A *Post-hoc* Analysis of Intravitreal Aflibercept-treated nAMD Patients from ARIES & ALTAIR: Predicting Treatment Intervals and Frequency for Aflibercept Treat-and-Extend Therapy Regimen using Machine Learning**

Matthias Gutfleisch,^1,2^* Britta Heimes-Bussmann,^1,2^* Sökmen Aydin,^2,3^ Ratko Petrovic,^3^ Alexander Loktyushin,^3^ Masahito Ohji,^4^ Kanji Takahashi,^5^ Annabelle A. Okada,^6^ Paula Scholz,^7^ Hossam Youssef,^8^ Ulrike Bauer-Steinhusen,^7^ Tobias Machewitz,^9^ Kai Rothaus,^1,2^ Albrecht Lommatzsch^1,2,10,11^

*Joint first authors

^1^Department of Ophthalmology, St Franziskus-Hospital, Münster, Germany

^2^M^3^ Macula Monitor Münster GmbH & Co KG, Münster, Germany

^3^deepeye Medical GmbH, Munich, Germany

^4^Shiga University of Medical Science, Seta Tsukinowa-cho, Otsu, Shiga, Japan

^5^Kansai Medical University, School of Medicine, Hirakata City, Osaka, Japan

^6^Kyorin University, School of Medicine, Mitaka-shi, Tokyo, Japan

^7^Bayer Vital GmbH, Leverkusen, Germany

^8^Bayer Middle East FZE, Dubai, United Arab Emirates

^9^Bayer AG, Berlin, Germany

^10^Department of Ophthalmology, University Duisburg-Essen, Essen, Germany

^11^Achim Wessing Institute of Ophthalmic Diagnostic, University Duisburg-Essen, Essen, Germany

**Corresponding author:** [matthias.gutfleisch@augen-franziskus.de](mailto:matthias.gutfleisch@augen-franziskus.de)

**CONTENTS**

**Online Resource 1:** ARIES and ALTAIR study designs

**Online Resource 2:** Criteria for maintenance or adjustment of treatment intervals in the ARIES and ALTAIR studies

**Online Resource 3:** Supplementary methods

**Online Resource 4:** SD OCT segmentation and processing

**Online Resource 5:** ARIES and ALTAIR study dataset device overview

**Online Resource 6: Performance of AI and study site biomarker assessment against the reading center**

**Online Resource 1: ARIES and ALTAIR study designs**


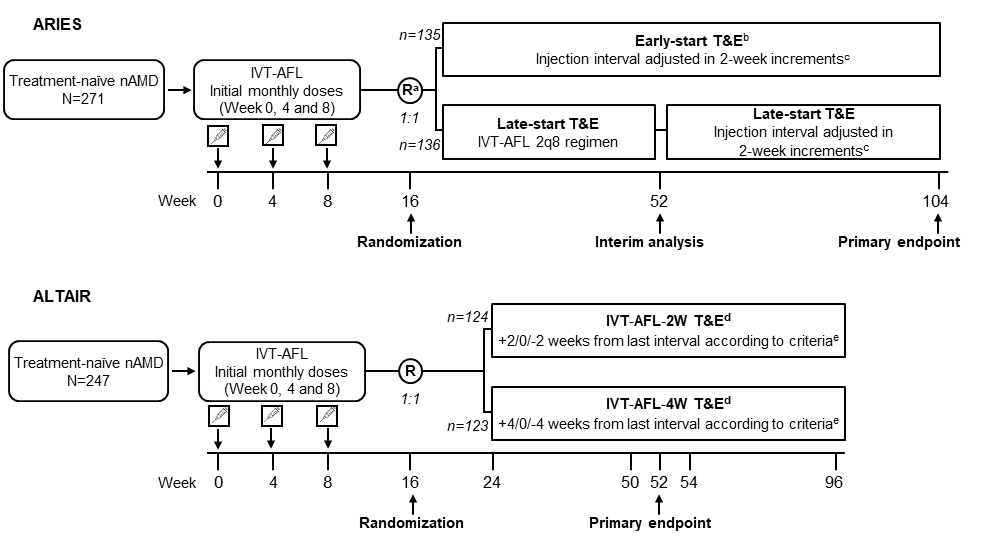


^a^ Patients were stratified based on visual outcomes from baseline to Week 16 (<8 letters or ≥8 letters gain in best-corrected visual acuity).

^b^ If no IRF and no SRF at Week 16, treatment could be extended from 8 to 12 weeks.

^c^ Injection interval could be extended to a maximum of 16 weeks.

^d^ Decision was based on pre-specified criteria with a maximum interval of 16 weeks.

^e^ Patients in the IVT-AFL-2W group could have their treatment interval increased or shortened by 2 weeks. Patients in the IVT‑AFL-4W group could have their treatment interval increased or shortened by 4 weeks. For patients in the IVT-AFL-4W group who had undergone interval shortening by 4 weeks, any subsequent interval extension or shortening was limited to 2 weeks.

2q8, 2 mg IVT-AFL every 8 weeks; 2W/4W, 2-/4-week adjustment; AMD, age-related macular degeneration; IRF, intraretinal fluid; IVT-AFL, intravitreal aflibercept; nAMD, neovascular AMD; R, randomization; SRF, subretinal fluid; T&E, treat-and-extend.

**Online Resource 2: Criteria for maintenance or adjustment of treatment intervals in the ARIES and ALTAIR studies**

**
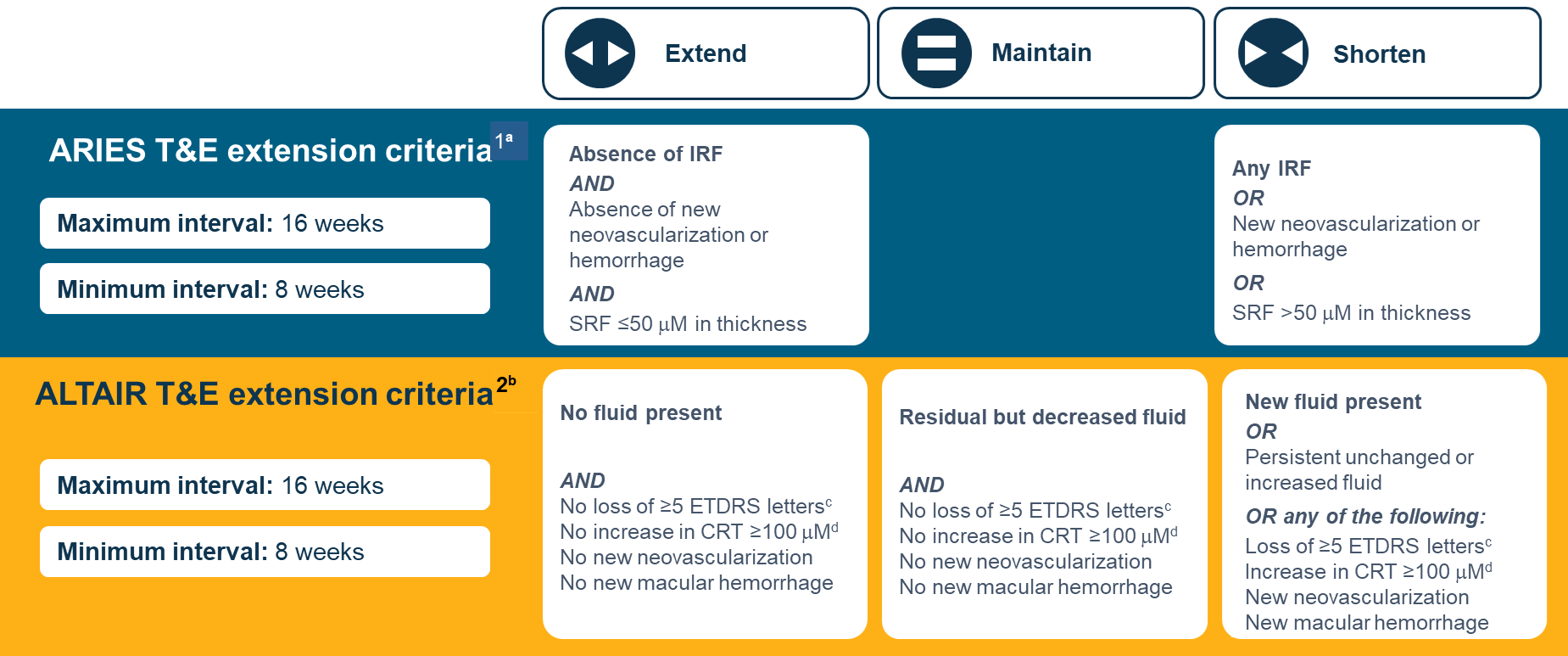
**

^a^ All outcomes were assessed by SD-OCT.

^b^ Fluid and CRT outcomes were assessed by SD-OCT.

^c^ From the last treatment visit, in conjunction with recurrent fluid on SD-OCT.

^d^Increase in CRT of ≥100 μm at central 1 mm compared with the lowest previous value by SD-OCT.
CRT, central retinal thickness; ETDRS, Early Treatment Diabetic Retinopathy Study; IRF, intraretinal fluid; SD-OCT, spectral-domain optical coherence tomography; SRF, subretinal fluid; T&E, treat and extend.

**References**

1. Mitchell P, Holz FG, Hykin P, Midena E, Souied E, Allmeier H, Lambrou G, Schmelter T, Wolf S (2021) Efficacy and safety of intravitreal efficacy using a treat- and-extend regimen for neovascular age-related macular degeneration: The ARIES study: A randomized clinical trial. Retina 41: 1911-1920 DOI 10.1097/IAE.0000000000003128
2. Ohji M, Takahashi K, Okada AA, Kobayashi M, Matsuda Y, Terano Y (2020) Efficacy and safety of intravitreal aflibercept treat-and-extend regimens in exudative age-related macular degeneration: 52- and 96-week findings from ALTAIR: A randomized controlled trial. Adv Ther 37: 1173-1187 DOI 10.1007/s12325-020-01236-x

**Online Resource 3: Supplementary methods**

***Data***

Prior to this, we had also exploratively tested other time point combinations (e.g. Baseline with Wk 8, and baseline with Wk 16) which resulted in poorer performances than with Wk 8 with Wk 16 scans. This observation aligned with our clinical practice, to compare consecutive OCTs.

***Development of AI models***

Training from scratch involves initializing a model with random weights and training it entirely on the provided dataset. Transfer learning uses a pre-trained model that has been trained on a larger dataset and fine-tunes its weights on a new usually much smaller dataset, leveraging knowledge gained from previous training to improve performance, compared to training from scratch.

***Data preprocessing***

The first step was to ensure the same field of view (FoV) for each B-scan by normalizing the pixel-distances of the height and width to the same reference size and then cropping it to the reference FoV (height = 1.92 mm; width = 5.8 mm) corresponding to 496x512 pixels. Thereafter, the region of interest (ROI) was masked by setting the region outside the area between the ILM and the choroid to zero pixels, as described in Gutfleisch et al. [1]. Subsequently, contrast-limited adaptive histogram equalization (CLAHE) was applied for contrast enhancement. Because the position of the ROI in each B-scan can differ both between scans within an acquisition and across acquisitions and manufacturers, each B-scan was aligned by applying shear based on the RPE endpoints. Then, axial direction translation was applied to a fixed position. Finally, each B-scan was resized to 256x256 pixels. In the case of scans acquired using Zeiss devices, the averaging was performed over the window of two neighboring slices to improve the signal-to-noise ratio. This was only performed for ROI detection rather than for subsequent steps.

***Deep learning model architecture***

Time-distributed convolutional neural network (CNN) was applied to extract the feature vector representation from each of the SD-OCT scans, passing the resulting feature vectors to the long short-term memory (LSTM) for the sequence learning of the time series mentioned above. LSTM is a well-established class of deep learning models used to process sequential data. In this project, only SD-OCTs from the upload phase were used for predictions. To address overfitting on this small dataset, the generated architecture is more compact. Instead of three convolutional blocks, as in [1], this architecture consists of two convolutional blocks. The number of feature maps is 8 in the first convolutional block and 32 in the second block, the kernel size is 5x5x5 and 1x3x3, respectively, followed by a global average pooling. Each convolutional block is composed of a sequence of a 3D convolutional layer, rectified linear unit activation, batch-normalization, and a 3D max pooling layer. The time-distributed output of global average pooling serves as an input to the LSTM layer with 32 hidden cells outputting only the last hidden cell with activated internal dropout-rate and a recurrent dropout rate, both of 0.5 (mitigating overfitting). The hyperbolic tangent is applied as the activation function. The output of the LSTM layer is connected to a fully connected layer with 32 units, and a dropout layer with a dropout rate of 0.5 (mitigating overfitting) concluding to a final soft max layer for the two-class prediction problem. The input size for our 3D-Architecture is 2x28x52x72 consisting of two consecutive SD-OCTs, each with 28 segmented B-scans containing only masks of IRF, SRF, and the layers ILM, IPL, OPL and RPE, with a height and width of 52x72 pixels.

***Model training for deep learning (ARIES)***

To prevent data leakage in each iteration, all data (clinical data and OCT) related to a patient appeared strictly in one subset only. As the input dataset was small, the 5-fold cross-validation was repeated 5x with random shuffling, resulting in 25 runs to obtain more reliable (with respect to statistical fluctuations) measures of the model performance.

Both models (transfer learning and training from scratch) were trained using Nadam optimizer [2], with a learning rate of 0.0001 with categorical cross entropy as the loss function. The batch size was set to 8. In the case of transfer learning, the weights of the PRN model mentioned above were used as initial weights. Depending on the experiment, either all layers or some of the convolutional blocks were frozen, and the remaining layers were trained. In Experiment 1, all layers were retrained; in the other experiments, the convolutional layers were frozen, and only the LSTM layer and the fully connected layers were retrained. In this study, we used 3D CNN over 2D CNN topologies, to capture spatial contexts in the B-scans dimension. Keras [3] served as the deep learning framework using TensorFlow [4] as the backend. The experiments were conducted on a dedicated machine running Ubuntu Server 20.04 and equipped with two linked graphics processing units (Nvidia GeForce Titan RTX, NVIDIA Corporation, Santa Clara, USA).

***Model training using logistic regression (ALTAIR)***

In this approach, deepeye® Research biomarker segmentation models [1] were used to predict the presence or absence of SRF and IRF at Wk 8 and Wk 16 and SRF/IRF binary prediction identifiers were merged into a single SRF/IRF variable. Logistic regression (regularization coefficient = 1000) was used to predict the endpoints in the experiments based on two scalars (SRF/IRF at Wk 8 and Wk 16) and 5-fold cross-validation was also performed to compensate for the small sample size.

***Experiments***

***Experiment 1*: *Prediction of the first treatment interval decision after treatment initiation***

In this case, the number of observed interval extension decisions in the first four visits following treatment initiation (i.e. Visits 4, 5, 6, and 7) would be used to determine the predicted interval decisions at Visit 7. This decision would then be extrapolated to a recommendation at Visit 4. The ground truth for assigning treatment intervals at Visit 7 was thus defined as follows: The assigned treatment interval at Visit 7 was defined as ‘short’ or ‘long’ if <3 or ≥3 (3 or 4) extension decisions, respectively, were taken at Visits 4, 5, 6, and 7, as illustrated below**:**

**Ground truth scenarios determining long and short intervals at Visit 7**

**
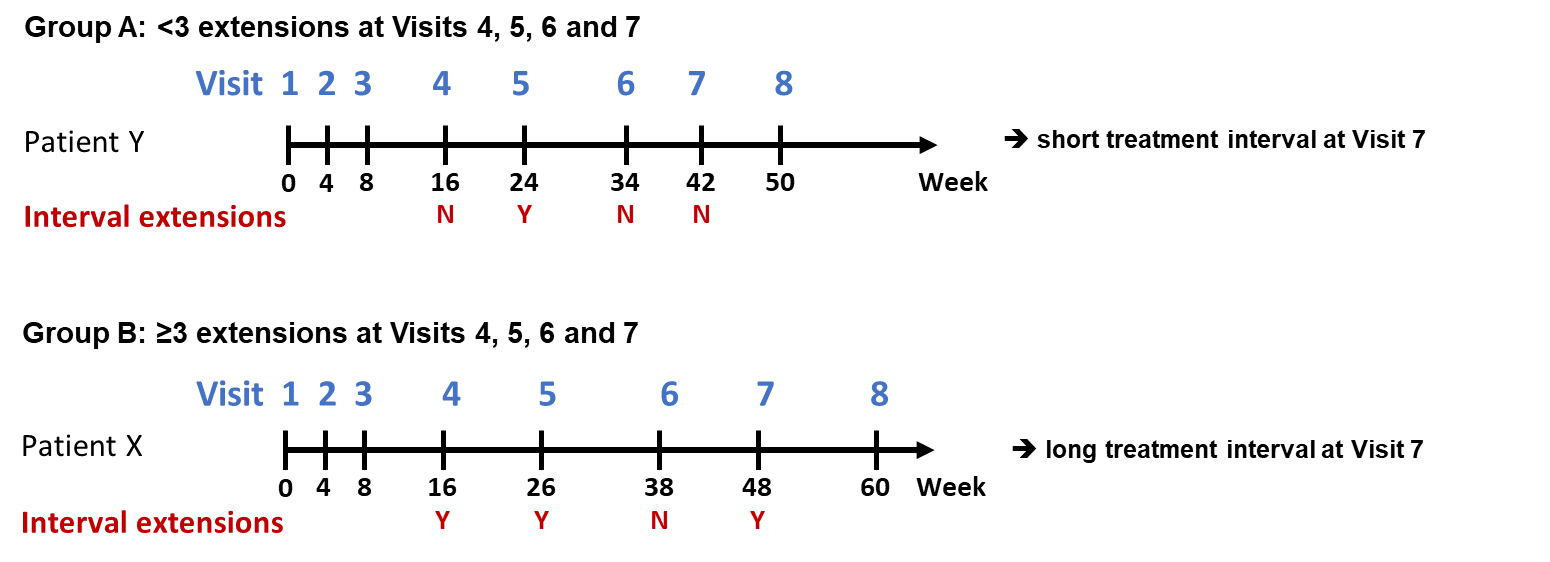
**

Patients in ARIES and ALTAIR were stratified into two groups with predicted short or long intervals at Visit 7 (Group A and B, respectively) and then tabulated against their actual observed treatment interval at Visit 7 (see below).

**Distribution of observed injection intervals at Visit 7 in patients from ARIES and ALTAIR**

|  | **Observed treatment interval at Visit 7 (weeks)** | | | | | |
| --- | --- | --- | --- | --- | --- | --- |
|  | ≤7 | 8–9 | 10–11 | 12–13 | 14–15 | ≥16 |
| **ARIES** | | | | | | |
| **Group A (n=72)**  **Predicted short interval at Visit 7** | 16 | 40 | 11 | 5 | – | – |
| **Group B (n=36)**  **Predicted long interval at Visit 7** | 1 | 1 | – | 6 | 9 | 19 |
| **ALTAIR** | | | | | | |
| **Group A (n=36)**  **Predicted short interval at Visit 7** | 6 | 24 | 4 | 1 | 0 | 1 |
| **Group B (n=45)**  **Predicted long interval at Visit 7** | 0 | 4 | 3 | 4 | 14 | 20 |

Group A: <3 extension decisions taken at Visits 4, 5, 6, and 7.

Group B: 3 or 4 extension decisions taken in Visits 4, 5, 6, and 7.

Statistical distribution demonstrated that, for Group A, the short interval at Visit 7 would correspond to <12 weeks, and for Group B, the long interval would correspond to ≥12 weeks. In general, the observed treatment intervals results aligned with the ground truth definition; however, several outliers were observed in ARIES and ALTAIR (5 and 2 patients, respectively, in Group A with observed intervals of ≥12 weeks, and 2 and 7 patients, respectively, in Group B with observed intervals of <12 weeks). This may have been because some patients in Group A delayed their treatment appointment. In contrast, other patients in Group B may have required attention/treatment earlier than their recommended subsequent visit.

**References:**

1. Gutfleisch M, Heimes-Bussmann B, Aydin S, Faatz P, Kintzinger K, Spickermann L, Tieck J, Koch H, Oehlschläger J, Ziegler M, Pauleikhoff D, Lange C, Spital G, Lommatzsch A, Rothaus K (2022) Annotation von SD-OCT-Biomarkern bei nAMD zur Entwicklung erklärbarer KI-Modelle (XAI)DOG (German Society of Ophthalmology) Congress, Berlin.
2. Dozat T (2016) Incorporating Nesterov Momentum into Adam. 4th International Conference on Learning Representations. <https://openreview.net/pdf/OM0jvwB8jIp57ZJjtNEZ.pdf>. Accessed 1 Aug 2024.
3. Chollet F (2015) Keras, GitHub. <https://github.com/fchollet/keras>.
4. Abadi M, Agarwal A, Barham P, Brevdo E (2015) TensorFlow: Large-scale machine learning on heterogeneous systems. <https://arxiv.org/abs/1603.04467>. Accessed 1 Aug 2024.

**Online Resource 4: SD-OCT segmentation and processing**

a) Prediction of IRF and SRF and layer segmentation masks using the AI model


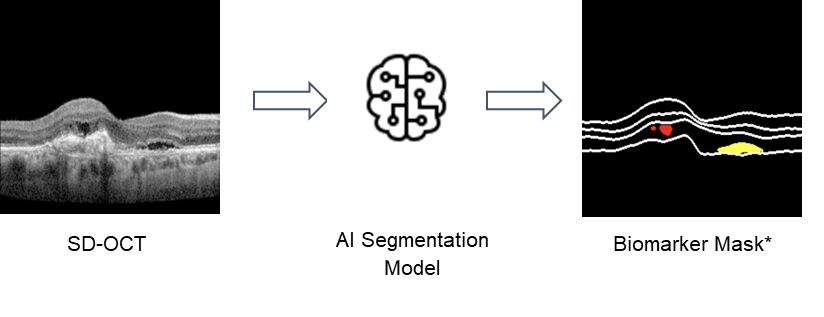


*Retinal layer, from top to bottom: ILM, IPL, OPL, RPE, IRF (red), and SRF (yellow)

b) AI training architecture


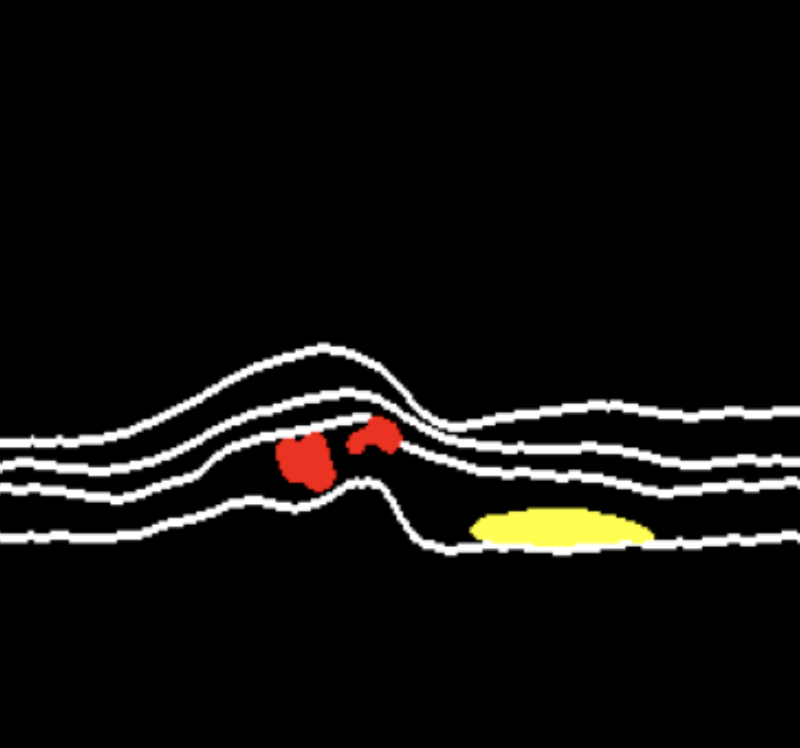

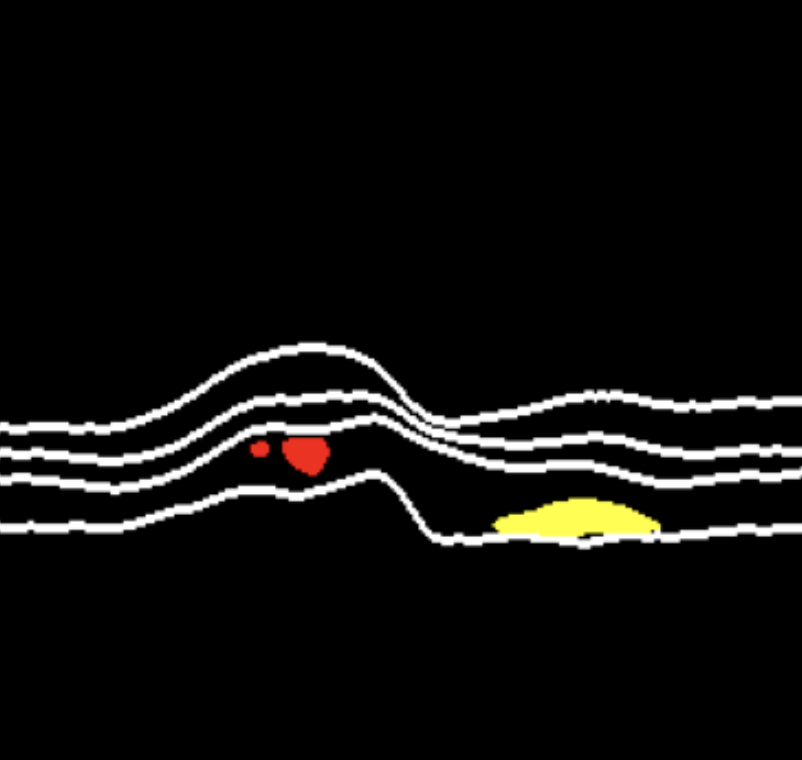


Time Distributed GAP

LSTM

Time Distributed Convolutional Blocks

Fully Connected Layer

Output

(class 0…n)

OCT (segmented) at Week 8

OCT (segmented) at Week 16

AI, artificial intelligence; GAP, global average pooling; ILM, inner limiting membrane; IPL, inner plexiform layer; IRF, intraretinal fluid; LSTM, long-short term memory; OCT, optical coherence tomography, OPL, outer plexiform layer; RPE, retinal pigment epithelium; SD-OCT, spectral-domain optical coherence tomography; SRF, subretinal fluid.

**Online Resource 5: ARIES and ALTAIR study dataset device overview**

| **ARIES** |  |  |
| --- | --- | --- |
| **Device type** | **Number of slices** | **Number of OCT scans** |
| Heidelberg Spectralis | 19 | 8 |
|  | 49 | 2229 |
| Carl Zeiss HD-OCT 4000 | 128 | 66 |
| Carl Zeiss HD-OCT 5000 | 128 | 209 |
|  | 200 | 58 |
| Topcon 3DOCT-2000 | 128 | 22 |
| Topcon Triton | 256 | 1 |
| **ALTAIR** |  |  |
| **Device type** | **Number of slices** | **Number of OCT scans** |
| Heidelberg Spectralis | 19 | 316 |
|  | 25 | 174 |
|  | 31 | 14 |
|  | 37 | 2 |
|  | 49 | 115 |
|  | 61 | 1 |
|  | 97 | 123 |
| Carl Zeiss HD-OCT 4000 | 128 | 130 |
|  | 200 | 55 |
| Carl Zeiss HD-OCT 5000 | 5 | 5 |
|  | 128 | 41 |
|  | 200 | 3 |
| Topcon 3DOCT-2000 | 128 | 37 |
| Topcon Triton | 256 | 327 |

OCT, optical coherence tomography

**Online Resource 6: Performance of AI and study site biomarker assessment against the reading center**

| **Comparison against reading center** | **IRF presence** | | **SRF height ≥50 µM** | | **SRF presence** | |
| --- | --- | --- | --- | --- | --- | --- |
|  | **AI** | **Study Site** | **AI** | **Study Site** | **AI** | **Study Site** |
| Balanced accuracy | 0.86 | 0.75 | 0.79 | 0.81 | 0.85 | N/A |
| Specificity | 0.86 | 0.63 | 0.60 | 0.87 | 0.85 | N/A |
| Sensitivity | 0.86 | 0.87 | 0.98 | 0.74 | 0.84 | N/A |

AI, artificial intelligence; IRF, intraretinal fluid; SRF, subretinal fluid.
